# Moral emotion processing in euthymic bipolar disorder: insights from network analysis

**DOI:** 10.1007/s00406-026-02344-5

**Published:** 2026-08-08

**Authors:** Marialaura Lussignoli, Giulia Cattarinussi, Alessandro Miola, Nicolò Trevisan, Silvia Valeggia, Renzo Manara, Fabio Sambataro

**Affiliations:** 1 Department of Neuroscience (DNS), University of Padova, Via Giustiniani, 2, 35128 Padua, Italy; 2 Padova Neuroscience Center, University of Padova, Padua, Italy; 3 Department of Psychological Medicine, Institute of Psychiatry, Psychology and Neuroscience, King’s College London, London, UK; 4 Department of Psychiatry & Psychology, Mayo Clinic, Rochester, MN USA; 5 Neuroradiology Unit, Department of Neurosciences, University of Padova, Padova, Italy

**Keywords:** Bipolar disorder, Facial emotion recognition, Anger, Disgust, Network analysis, Gray matter volume

## Abstract

**Background:**

Bipolar disorder (BD) is associated with facial emotion recognition (FER) deficits. Among these, impairments in recognizing moral emotions may be especially relevant to social dysfunction, but their relationship with brain structure remains poorly defined.

**Methods:**

In this cross-sectional case–control study, 48 euthymic adults with BD (BD-I, *n* = 20; BD-II, *n* = 28) and 45 sex- and age-matched healthy controls completed a computerized FER task and 3T T1-weighted MRI scan. FER efficiency (accuracy/response time) for facial moral (anger and disgust) emotion expressions and gray-matter volume (GMV) from 9 bilateral a priori fronto–limbic/salience regions were compared between groups. Group-specific psychometric networks (regularized partial-correlation, i.e. Gaussian graphical models; LASSO/EBIC) integrating FER and GMV nodes were estimated and compared using the Network Comparison Test.

**Results:**

Relative to BD-II and controls, BD-I showed lower FER efficiency, with poorer decoding of moral but not neutral facial expressions. Network structure differed between BD-II and controls, whereas global network strength was comparable across all pairwise group comparisons. Exploratory centrality analyses suggested a more frontally weighted configuration in BD-I, with the superior frontal gyrus among the more central nodes.

**Conclusion:**

In BD-I, persistently reduced ability to decode morally salient facial cues co-occurred with subtype-related differences in fronto–limbic structural covariance.

**Supplementary Information:**

The online version contains supplementary material available at https://doi.org/10.1007/s00406-026-02344-5.

## Introduction

Bipolar disorder (BD) is a severe psychiatric illness characterized by marked mood swings between manic, hypomanic, and depressive episodes. Beyond affective symptoms, growing evidence indicates that BD is associated with persistent deficits across several cognitive domains, including social cognition, defined as the capacity to understand and respond appropriately to social information [1]. Such impairments are observed even outside acute mood phases: euthymic individuals with BD continue to exhibit social-cognitive difficulties, suggesting that these deficits may represent trait-like endophenotypes of the disorder [2]. Moreover, these impairments hold clinical significance, as they correlate with poorer psychosocial functioning and lower quality of interpersonal relationships in BD [3]. Thus, BD entails not only fluctuations in mood but also enduring cognitive anomalies, particularly in affective cognition [4], which contribute to impaired social functioning.

Although BD type II (BD-II) has historically been viewed as a milder variant, recent studies have uncovered significant neurocognitive and clinical distinctions between the subtypes [5], leading some authors to propose conceptualizing them as a partially distinct syndrome [6]. For instance, neurocognitive performance is often more impaired in BD type I (BD-I) than in BD-II [5]. Whether such differences extend to domains of social cognition remains unclear. While an early meta-analysis found no significant overall subtype differences in social-cognitive performance [1, 7], more recent evidence points toward a heterogeneous picture: patients with BD-I sometimes display greater impairments in emotion processing tasks, whereas BD-II patients may show subtler challenges in social inference and intent attribution tasks [1]. These discrepancies underscore the need for targeted investigations into subtype-specific social-cognitive profiles.

Within social cognition, the ability to recognize facial emotional expressions, known as Facial Emotion Recognition (FER), is fundamental to everyday interactions. A wealth of studies has documented FER deficits in BD relative to healthy controls (HC) [8]. Converging evidence indicates that people with BD exhibit reduced accuracy in identifying faces expressing negative emotions (e.g., fear, sadness, disgust, anger). For example, even during euthymia, individuals with BD demonstrate particular difficulty recognizing disgust, while manic episodes further impair recognition of fear and other negative emotions [9]. Additionally, individuals with BD may misinterpret ambiguous or neutral facial expressions as negative, or confuse anger with other affective states [10]. These findings reveal a widespread alteration in decoding emotional signals in BD, particularly for socially salient negative emotions, which likely contributes to the interpersonal challenges commonly reported by these patients.

Among basic emotions, anger and disgust hold special significance as “moral” emotions [11]. Both often arise in response to violations of social norms or ethical values, signaling condemnation or disapproval of objectionable behavior [12, 13]. Social-psychological research demonstrates that moral disgust frequently co-occurs with anger when individuals witness norm violations: the facial expression of disgust in moral contexts typically carries an underlying component of wrath. In other words, “moral” disgust overlaps with anger in conveying negative moral judgments [11]. Accurate identification of anger and disgust in others’ faces is therefore essential for correctly interpreting the social and moral meaning of a situation. A deficit in this ability could lead individuals with BD to underestimate or misread the severity of others’ moral outrage, potentially impairing their social functioning. This conceptual link between emotional recognition and moral judgment motivates a focused investigation of individuals with BD’s capacity to recognize morally salient facial expressions and to explore whether such deficits relate to disruptions in the underlying neural circuitry.

Neurobiologically, in healthy individuals, moral-emotion and moral-judgment tasks engage a distributed cortico-limbic network [14, 15]. Early fMRI studies showed that stimuli evoking moral emotions recruit the amygdala together with orbital/medial prefrontal and superior temporal regions, while work on disgust-based moral appraisal further implicated frontal, temporal, and limbic circuits [16]. Meta-analytic evidence subsequently confirmed convergent involvement of the ventromedial/dorsomedial prefrontal cortex, temporo-parietal and posterior cingulate/precuneus regions, and the amygdala during moral cognition [14, 15, 17]. In keeping with this, the social-cognitive and emotional recognition impairments seen in BD have been linked to altered gray matter volume across prefrontal, salience-related (e.g., anterior cingulate and insular cortices), and limbic regions [18–20]. Importantly, these alterations may not be confined to isolated regions: structural covariance approaches capture how gray-matter morphology in distributed nodes varies together across individuals, providing an index of large-scale anatomical coordination. In euthymic BD, morphometry-based network analyses suggest that fronto–salience–limbic coordination patterns are reorganized and may relate to individual differences in emotion recognition [21–23]. This structural-network perspective provides a coherent framework to test whether subtype-specific differences in moral emotion decoding map onto distinct patterns of fronto–limbic anatomical coordination.

Taken together, cognitive and neural findings in BD point to a tightly intertwined relationship between emotion-processing deficits (e.g., poor FER and theory-of-mind impairments) and fronto-limbic dysconnectivity. To fully understand moral emotion processing in BD, it is thus essential to integrate behavioral performance measures with neuroanatomical data. Psychometric network analysis offers a powerful multivariate methodology to this end. Unlike traditional pairwise correlation approaches, network models can capture the complex web of associations among multiple variables, identifying key nodes and the overall structure of interrelationships [24]. In psychopathology and neuroscience research, such methods [25] have illuminated pathways of pathological interaction that might be overlooked by univariate analyses. Applied to BD, network analysis of FER performance and brain morphometry could reveal distinct connectivity patterns, highlighting how structural variations in limbic and frontal regions relate collectively to deficits in recognizing moral emotions. A recent study employing this approach for sadness recognition in euthymic BD found an altered network topology: patients displayed decoupling among frontal, insular, and occipital regions in gray-matter correlations, coupled with a heightened central role of the amygdala in linking brain structure to FER accuracy [23]. These findings suggest that BD may rely disproportionately on limbic nodes, reflecting impaired cortical regulation of emotion [26].

Building on this theoretical framework, the present study examines recognition of moral facial expressions (specifically anger and disgust) in euthymic patients with BD-I and BD-II compared with HC. We aim to assess subtype-specific differences in FER accuracy for moral emotions, and to explore how performance on these tasks relates to gray-matter volumes in key fronto-limbic regions using a network-analytic approach. This integrative methodology, which combines behavioral and neuroanatomical measures, aims to clarify the neurocognitive mechanisms underlying moral emotion recognition in BD and to identify potential distinctions between BD-I and BD-II. Clarifying whether moral-emotion recognition and its structural substrates differ between BD-I and BD-II is clinically relevant, given the partly distinct clinical and neurobiological profiles of the two subtypes [27]. Subtype-specific social-cognitive profiles could refine prognosis and guide targeted interventions, including social‑cognitive remediation programs that have shown preliminary benefits in bipolar disorder [28], and may help explain the differential psychosocial outcomes reported across subtypes. We hypothesized that, relative to HC, individuals with BD would exhibit significant alterations in moral emotion recognition and in fronto–limbic structural covariance, with distinct patterns across BD-I and BD-II.

## Materials and methods

### Participants

We enrolled 51 adults with BD and 45 age- and sex-matched HC. All BD diagnoses were confirmed with the Structured Clinical Interview for DSM-5 - Patient Edition (SCID-5); patients were required to be clinically stable and on a stable pharmacological regimen for ≥ 1 month. HC were screened to exclude current or past psychiatric disorders, a personal or family history of severe psychosis, and ongoing pharmacological treatment (except oral contraceptives in women). Common exclusion criteria for both groups were age outside 18–65 years, substance dependence or significant misuse within the previous six months, prior traumatic brain injury with loss of consciousness, major medical/neurological disorders, and intellectual disability. During data collection, three BD participants were withdrawn: one experienced a panic attack during MRI acquisition, and two exhibited vascular abnormalities on imaging. Consequently, the final sample comprised 48 participants with BD (20 with BD-I and 28 with BD-II) and 45 HC.

Written informed consent was obtained from all participants for the anonymous, aggregate use of their data. Under local IRB policy, no further consent was required for the retrospective, naturalistic review of medical records. This study adhered to the ethical principles of the Declaration of Helsinki (1964), as updated by the 75th WMA General Assembly in Helsinki (World Medical Association, 2025) [29].

### Clinical assessment

To assess the severity of affective symptoms, several standardized rating scales were employed: the Montgomery–Åsberg Depression Rating Scale (MADRS) [30], the 17-item Hamilton Rating Scale for Depression (HAM-D) [31], the Hamilton Anxiety Rating Scale (HAM-A) [32], and the Young Mania Rating Scale (YMRS) [33]. Psychotic features were evaluated using the Positive and Negative Syndrome Scale (PANSS) [34]. Overall psychosocial functioning was measured using the Global Assessment of Functioning (GAF) scale [35].

In addition, a comprehensive clinical history related to mood disorders was collected. This included information on illness duration, age at onset, the total number of lifetime affective episodes (including depressive, manic, hypomanic, and mixed episodes). Data regarding current pharmacological treatments were recorded using the Defined Daily Dose metric [36], along with serum lithium concentrations and the duration of lithium therapy.

### Facial emotion recognition (FER) task

Emotional processing was assessed using a FER task [37, 38] administered via PEBL software (http://pebl.sourceforge.net/). The task consists of showing 140 facial stimuli expressing various emotions in a pseudo-randomized sequence: sadness (*n* = 40), disgust (*n* = 40), anger (*n* = 40), and neutral expressions (*n* = 20). Each face was presented for 2000 msec, and participants were required to promptly identify the displayed emotion by selecting the appropriate label using a touchscreen interface. Both response accuracy and reaction time were recorded. To assess the efficiency of emotional recognition, defined as weighting the overall accuracy by the speed of the responses to the task, a performance index was calculated by dividing the percentage of correct responses by the mean reaction time [39], computed both globally (FER-total) and for each individual emotion (FER-sadness, FER-anger, FER-disgust, FER-neutral). This efficiency index was chosen a priori because it integrates speed and accuracy, mitigates speed–accuracy trade-offs, and reduces the ceiling effects typical of accuracy-only FER measures. Moral FER (FER‑moral) was computed as the unweighted arithmetic mean of FER‑anger and FER‑disgust (equal weights 0.5/0.5), following the notion of anger and disgust as moral emotions [11].

Given our strong a priori hypothesis on the processing of morally salient emotional cues, our analyses focused on FER performance for moral (FER-moral) and neutral (FER-neutral) expressions, the latter serving as a non-moral control condition [40]. Although sadness was administered as part of the standardized task, it was excluded a priori from the present analyses because it is not classified as a moral emotion within the adopted framework [12]. We selected an efficiency index (percent accuracy divided by mean reaction time) a priori because it integrates speed and accuracy, mitigates speed–accuracy trade-offs, and reduces ceiling effects typical of accuracy-only FER measures in high-functioning euthymic samples [39]; accuracy and reaction-time results are additionally reported in the Supplementary Material.

### Image acquisition

High-resolution structural brain images were obtained using a 3 Tesla Philips Ingenia MRI scanner equipped with a 32-channel quadrature head coil. Each participant underwent a whole-brain 3D T1-weighted magnetization-prepared rapid gradient-echo sequence, acquired in the sagittal plane. Imaging parameters were as follows: TR/TE = 6676 ms/3 ms; field of view (FOV) = 240 mm; flip angle = 8°; resolution = 1.0 × 1.0 × 1.0 mm³; number of slices = 181. All scans were reviewed by an experienced neuroradiologist (RM) to exclude any brain abnormalities.

### Voxel-based morphometry (VBM)

Structural MRI data were processed using the Computational Anatomy Toolbox for SPM (CAT12; http://www.neuro.uni-jena.de/cat/) within the Statistical Parametric Mapping framework (SPM12; http://www.fil.ion.ucl.ac.uk/spm/software/spm12/). After conducting quality checks to rule out artifacts such as motion, ghosting, or image distortions, each T1-weighted image was reoriented to the ACPC line. The images were then spatially normalized and segmented into gray matter (GM), white matter (WM), and cerebrospinal fluid (CSF) using a maximum a posteriori approach. Following preprocessing, normalized and modulated GM and WM images were smoothed with an 8 mm full-width at half-maximum (FWHM) Gaussian kernel. An absolute threshold of 0.2 was applied to mask out non-brain voxels. All volumetric images underwent both visual inspection and statistical quality control to assess intersubject homogeneity and to detect any potential processing artifacts. The analysis focused solely on gray matter volumes (GMVs), in line with the study’s interest in structural covariance. Total intracranial volume (TIV) was estimated to account for individual differences in brain size. GMVs were extracted for nine bilateral regions of interest (ROIs) based on the n30r83 version of the Hammersmith probabilistic brain atlas (http://brain-development.org/brain-atlases/adult-brain-maximum-probability-map-hammersmith-atlas-n30r83-in-mni-space/). The number and identity of the regions were defined a priori, to maximise comparability with prior structural-covariance work on FER in euthymic BD that used the same atlas and regions [23] and to limit network complexity relative to the sample size. Based on neuroimaging evidence supporting bilateral involvement in the processing of anger and disgust the ROIs were drawn bilaterally [41]. The selected brain regions included those implicated in the processing of facial affect recognition as in: the amygdala [42–44], hippocampus [43, 45], insula [43, 46], anterior cingulate cortex [42, 43, 47], orbitofrontal cortex, fusiform gyrus [42, 43], superior frontal gyrus, middle frontal gyrus, and inferior frontal gyrus [42, 48, 49].

### Statistical analysis

Group differences in socio-demographic and clinical characteristics were examined using chi-square tests for categorical variables and one-way analysis of variance (ANOVA) for continuous variables. When omnibus tests reached statistical significance, pairwise comparisons were conducted using chi-square tests for categorical data and Tukey’s post hoc tests for continuous measures. The index of moral emotion recognition performance (FER-moral), was computed as the average of each participant’s FER-anger and FER-disgust scores [11]. Comparisons of FER-total, FER-moral, and FER-neutral performance across the three diagnostic groups (BD-I, BD-II, and HC) were conducted using ANOVA and repeated-measures ANOVA, respectively. Pairwise comparisons between FER-moral and FER-neutral scores within each diagnostic group were performed, applying Tukey’s correction for multiple testing. In addition to the efficiency index, response accuracy (percentage of correct responses) and mean reaction times were compared across the three groups for moral and neutral expressions using one-way ANOVA (Supplementary Table S6). Voxel-wise comparisons of gray-matter volumes (GMV) across diagnostic groups were carried out using a general linear model (GLM), with TIV and age included as covariates. Post hoc pairwise t-tests were used to identify specific group differences. Within each diagnostic group, associations between FER scores and clinical variables were assessed using Pearson’s or Spearman’s correlation coefficients, as appropriate based on data distribution. For morphometry, GMV from nine bilateral ROIs was analyzed with GLMs including age, TIV, and sex (education additionally included where available) as covariates; false discovery rate correction (Benjamini–Hochberg, q < 0.05) was applied to ROI-wise GMV comparisons. Analyses other than the ROI-wise GMV comparisons (pairwise FER comparisons, edge-level network comparisons, centrality, and brain–behaviour correlations) are reported with both uncorrected and Benjamini–Hochberg-corrected p-values (Supplementary Material). All statistical analyses were performed using JAMOVI (Version 2.6.17; https://www.jamovi.org) and R version 4.3 (https://www.rstudio.com/). Statistical significance was defined as *p* < 0.05.

### Network analysis

To explore the relationship between FER-moral scores and GMV across groups (BD-I, BD-II, and HC), we conducted a set of network analyses.

For each group, a network was constructed using 11 nodes: two representing FER performance (FER-moral and FER-neutral, the latter being a control index) and nine representing regional GMV values. Edges between nodes represented partial correlations.

All network analyses were conducted in R version 4.3 [50]. Network estimation and visualization were performed using the qgraph package [51], while accuracy was assessed with the bootnet package [52]. Spearman’s correlations were used throughout. Networks were regularized using the Least Absolute Shrinkage and Selection Operator (LASSO) method [53, 54], and sparsity was controlled via the Extended Bayesian Information Criterion (EBIC) with a gamma (γ) hyperparameter set at 0.25, based on prior simulation results (netSimulator). Network centrality was evaluated using standard indices [24] (strength, closeness and betweenness). Group differences in global network structure and overall connectivity (e.g., average strength of all edges) were assessed using the Network Comparison Test (NCT), a two-tailed independent-groups permutation test with 5,000 iterations [55].

We evaluated two key statistics: *M*, the Network Structure invariance statistic, which is the sum of absolute differences across all corresponding edge weights, and *S*, the Global-strength invariance statistic (the difference in the total absolute edge weight of each network) [52]. A significance threshold of *p* < 0.05 was used for all tests. Furthermore, to evaluate the stability and reliability of the network metrics, bootstrapping procedures (5,000 samples) were employed to compute 95% confidence intervals for edge weights and to estimate the centrality stability coefficient (CS-coefficient). The CS-coefficient quantifies the proportion of the sample that can be randomly dropped while maintaining a correlation of at least 0.70 with the original centrality metrics at 95% confidence. A CS-coefficient above 0.50 is considered acceptable [25].

## Results

### Sociodemographic and clinical characteristics of study participants

Sociodemographic and clinical characteristics are reported in Table 1. There were no significant differences among groups in demographics (age, sex), and clinical variables (illness duration, HAM-D, HAM-A, MADRS, and YMRS scores) with all p-values > 0.1. Patients with BD-I exhibited lower GAF scores (*p* = 0.004), and fewer past depressive (*p* = 0.033) and hypomanic (*p* = 0.004) episodes compared to those with BD-II. Conversely, medication exposure differed when expressed as the number of drugs prescribed: patients with BD-II reported significantly greater antidepressant (*p* < 0.001) and lower antipsychotic treatment (*p* = 0.007) than BD-I; no significant between-group differences emerged for anticonvulsants, lithium use, or current plasma lithium levels (all p-values > 0.1). Finally, none of the recruited BD-I and BD-II patients had psychiatric comorbidities.

**Table 1:** Socio-demographic and clinical characteristics of the sample

Characteristics	BD-I (*N* = 20)	BD-II (*N* = 28)	HC (*N* = 45)	F or χ²	*p*
Age (years), mean ± SD	45.5 ± 12.6	38.9 ± 12.6	40.1 ± 12.8	1.745	0.186
Males, n (%)	13 (65.0)	19 (67.9)	23 (51.1)	0.589	0.745
Duration of illness (years), mean ± SD	16.6 ± 10.1	12.8 ± 10.7	NA	1.239	0.222
Childhood onset, n (%)	3 (15.0)	11 (39.3)	NA	−1.852	0.070
Previous psychotic symptoms, n (%)	11 (55.0)	10 (36.0)	NA	4.899	< 0.001
Family history for BD, n (%)	15 (75.0)	18 (64.3)	NA	0.778	0.441
*Number of past episodes*					
Depressive (*N* = 0/1/≥ 2)	6/0/12	1/2/19	NA	6.820	0.033
Manic (*N* = 0/1/≥ 2)	2/8/8	20/0/0	NA	30.707	< 0.001
Hypomanic (*N* = 0/1/2+)	11/3/4	2/6/12	NA	11.156	0.004
Mixed (*N* = 0/1/≥ 2)	14/4/0	20/0/0	NA	5.200	0.023
HAMD, mean ± SD	2.83 ± 5.52	1.69 ± 2.25	NA	0.900	0.374
HAMA, mean ± SD	3.33 ± 6.21	1.45 ± 1.90	NA	1.389	0.173
MADRS, mean ± SD	3.67 ± 7.23	2.17 ± 4.24	NA	0.827	0.413
YMRS, mean ± SD	3.00 ± 6.70	1.09 ± 2.44	NA	1.248	0.220
GAF, mean ± SD	65.00 ± 23.37	80.58 ± 8.41	NA	−3.076	0.004
*Current pharmacotherapy*					
Antidepressants, n (%)	6 (30.0)	22 (78.6)	NA	−3.365	< 0.001
Antipsychotics, n (%)	15 (75.0)	10 (35.7)	NA	2.686	0.007
Anticonvulsants, n (%)	6 (30.0)	4 (14.3)	NA	1.322	0.187
Lithium, n (%)	19 (95.0)	28 (100)	NA	−1.196	0.230
Lithium treatment duration (months), mean ± SD	89.5 ± 118.9	28.3 ± 40.6	NA	2.531	0.015
Lithium plasma level (mmol/L), mean ± SD	0.550 ± 0.270	0.522 ± 0.170	NA	0.427	0.672

An ANOVA and a chi-square test were performed to compare age and sex among groups. Two-sample t-tests and chi-square tests were performed for continuous and categorical variables, respectively, when only two groups were compared

*HAMD* Hamilton rating scale for depression, *HAMA* Hamilton rating scale for anxiety, *MADRS* Montgomery–Åsberg depression rating scale, *NA* Not applicable, *YMRS* Young mania rating scale, *GAF* Global assessment of functioning, *SD* Standard deviation, *BD-I* Bipolar disorder type I, *BD-II* Bipolar disorder type II, *HC* Healthy controls

### FER task

**Fig. 1:**
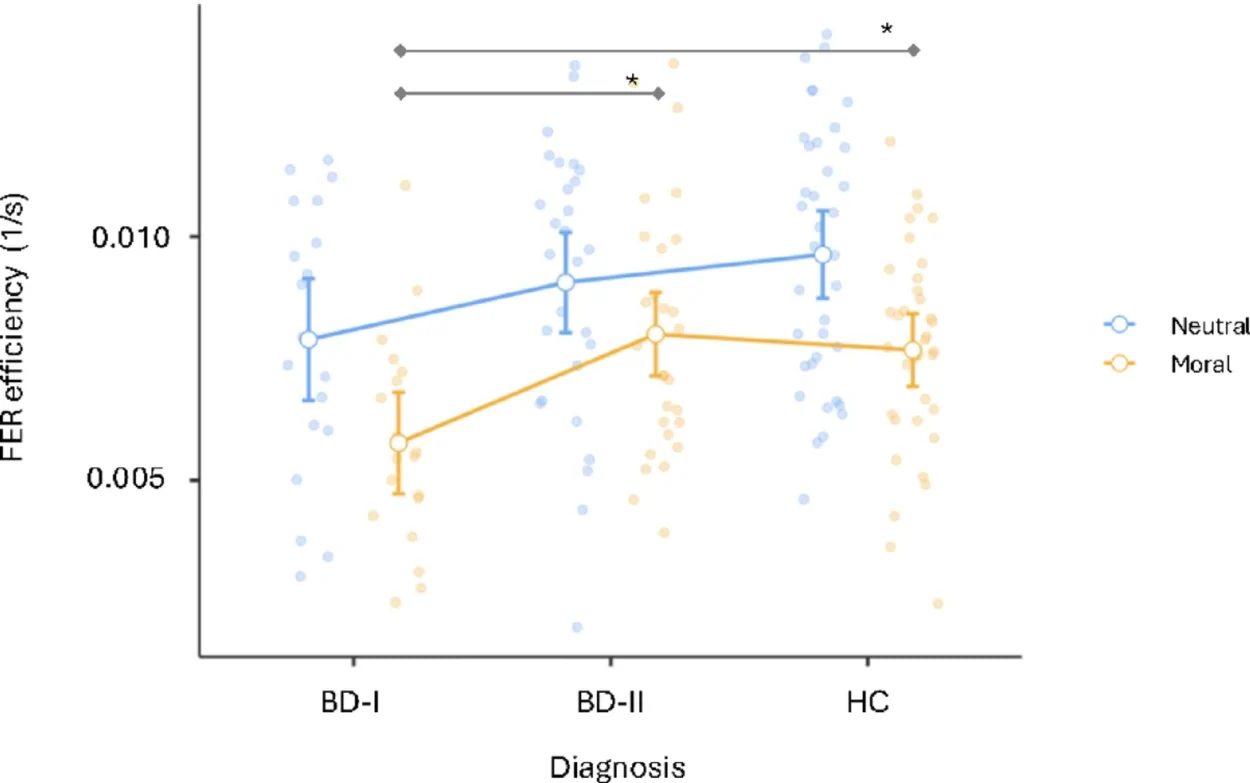
Facial expression recognition (FER) performance for moral versus neutral expressions across diagnostic groups (BD-I, BD-II, HC). Relative to BD-II and HC, BD-I showed reduced FER during the presentation of moral emotions, whereas performance for neutral expressions was comparatively preserved; BD-II displayed similar performance across emotions. Each dot represents an individual participant; solid circles and error bars indicate group means and 95% confidence intervals for each emotion within each diagnosis (colors follow the legend). FER performance scores were computed as the ratio of percent accuracy to mean reaction time. Asterisks (*) denote Tukey-corrected between-group comparisons within the Moral condition (*p* < 0.05: BD-I vs. BD-II; BD-I vs. HC). Abbreviations: FER, facial emotion recognition; BD-I, bipolar disorder type I; BD-II, bipolar disorder type II; HC, healthy controls

There was a main effect of Diagnosis on FER‑total score, F(2, 81) = 6.51, *p* = 0.002 (BD‑I < BD‑II, Tukey *p* = 0.009; BD‑I < HC, *p* = 0.003; BD‑II ≈ HC, *p* = 0.963). There was also a main effect of Emotion (moral vs. neutral), F(1, 81) = 20.534, *p* < 0.001. The Diagnosis × Emotion interaction was not significant, F(2, 81) = 0.774, *p* = 0.464. Planned comparisons showed that BD‑I performed worse on FER‑moral than BD‑II (*p* = 0.018) and HC (*p* = 0.045), whereas BD-II and HC did not differ (*p* = 0.993). By contrast, FER‑neutral did not differ between groups (BD‑I vs. BD‑II, *p* = 0.703; BD‑I vs. HC, *p* = 0.224; BD‑II vs. HC, *p* = 0.960) (Fig. 1). Consistent with the efficiency findings, overall accuracy was lower in BD-I than in BD-II and HC (F(2,76) = 4.43, *p* = 0.015), and reaction times for moral expressions were significantly longer in BD-I (F(2,76) = 6.21, *p* = 0.003), whereas accuracy for moral expressions did not differ significantly across groups (F(2,76) = 2.56, *p* = 0.084) (Supplementary Table S6). After adjusting for sex and education, the group effect on FER-moral remained significant (F(2,82) = 4.42, *p* = 0.015; BD-I < BD-II *p* = 0.008, BD-I < HC *p* = 0.011; BD-II ≈ HC) and persisted after additionally adjusting for age (*p* = 0.037).

Antipsychotic Defined Daily Dose (DDD) was higher in BD-I than BD-II (0.95 ± 0.94 vs. 0.17 ± 0.37, *p* = 0.030). Within patients, antipsychotic DDD correlated negatively with FER-moral (Spearman ρ = −0.42, *p* = 0.028), whereas antidepressant DDD (ρ = +0.38, *p* = 0.038) and lithium (ρ = +0.44, *p* = 0.018) correlated positively. In a sensitivity analysis, in a smaller sample with these data (*n* = 28), the BD-I vs. BD-II difference in FER-moral (unadjusted *p* = 0.003) was no longer significant after adjusting for antipsychotic and antidepressant DDD (*p* = 0.82) (Supplementary Table S5).

### Network analyses

**Fig. 2:**
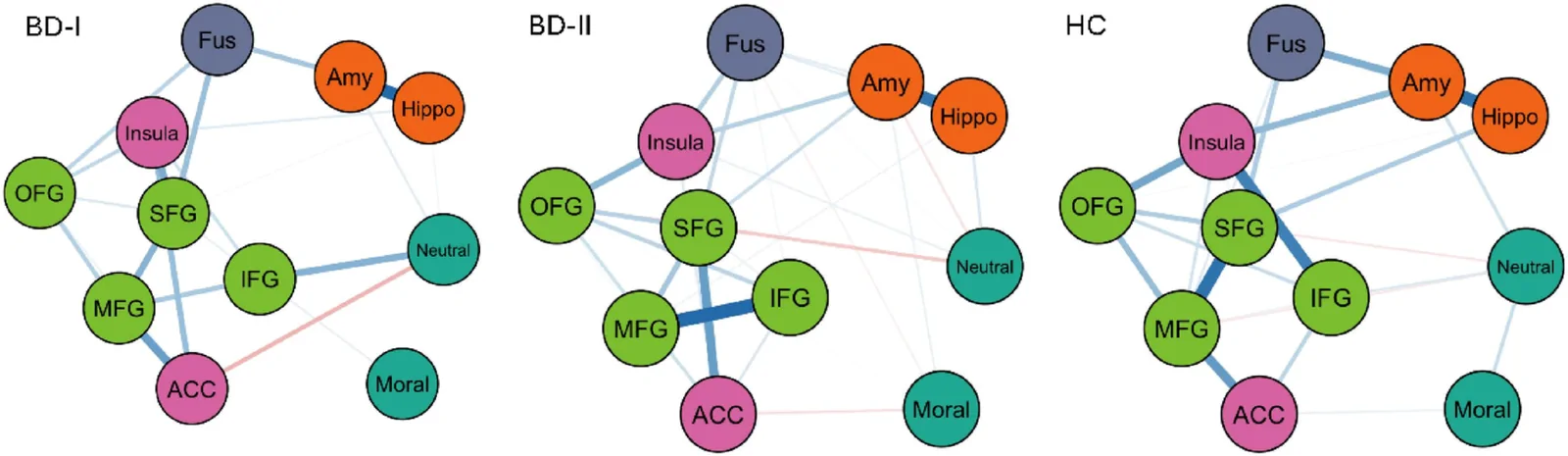
Networks of facial emotion recognition and brain morphometry of the regions implicated in facial emotion recognition (FER) for each diagnostic group (BD-I, BD-II, HC) Partial-correlation networks relating FER performance to gray-matter volume (GMV) in regions involved in facial-emotion processing, shown separately for BD-I, BD-II, and HC. Node colors: green, prefrontal cortex (OFG, IFG, MFG, SFG); magenta, salience (insula, ACC); blue-gray, fusiform gyrus; orange, limbic (amygdala, hippocampus); teal, behavioral variables (FER-moral, FER-neutral). Edge thickness depicts the strength of the partial association (controlling for the other nodes); edge color indicates the sign (blue = positive, red = negative). The node layout is held constant across panels to facilitate visual comparison. Abbreviations: FER, facial emotion recognition; GMV, gray-matter volume; BD-I/BD-II, bipolar disorder type I/II; HC, healthy controls; OFG, orbitofrontal gyrus; IFG, inferior frontal gyrus; MFG, middle frontal gyrus; SFG, superior frontal gyrus; ACC, anterior cingulate cortex; Insula, insular cortex; Fus, fusiform gyrus; Amy, amygdala; Hippo, hippocampus; Moral/Neutral, behavioral task conditions

The network analyses revealed a significant difference in overall network structure between BD-II and HC (M = 0.550, *p* = 0.009). In contrast, no significant differences were found between BD-I and HC (M = 0.297, *p* = 0.830) or between BD-I and BD-II (M = 0.409, *p* = 0.481). Regarding global network strength, no significant differences were observed among groups (BD-I vs. BD-II: S = 0.130, *p* = 0.910; BD-I vs. HC: S = 0.149, *p* = 0.879; BD-II vs. HC: S = 0.280, *p* = 0.672) (Fig. 2). The full partial-correlation edge weights for each group, together with their bootstrapped 95% confidence intervals, are reported in Supplementary Table S3.

As for the brain–behavior correlations, the partial Spearman association between FER‑neutral and amygdala GMV was near‑zero in BD‑I (r_p = 0.025; 95% CI − 0.61 to + 0.65), negative in BD‑II (r_p = − 0.586; 95% CI − 0.82 to − 0.18), and weakly positive in HC (r_p = 0.158; 95% CI − 0.21 to + 0.49). The association was greater in BD‑I and HC than in BD‑II, whereas BD‑I and HC were similar. At the edge level (independent-groups NCT), several fronto–limbic GMV edges differed between groups at uncorrected thresholds (full uncorrected and FDR-corrected values in Supplementary Table S2). After Benjamini–Hochberg correction across all 55 tested edges per comparison, only the Insula–IFG difference in the BD-II vs. HC comparison remained significant (p_uncorrected = 0.0006, p_FDR = 0.033); all BD-I comparisons were non-significant after correction.

**Fig. 3:**
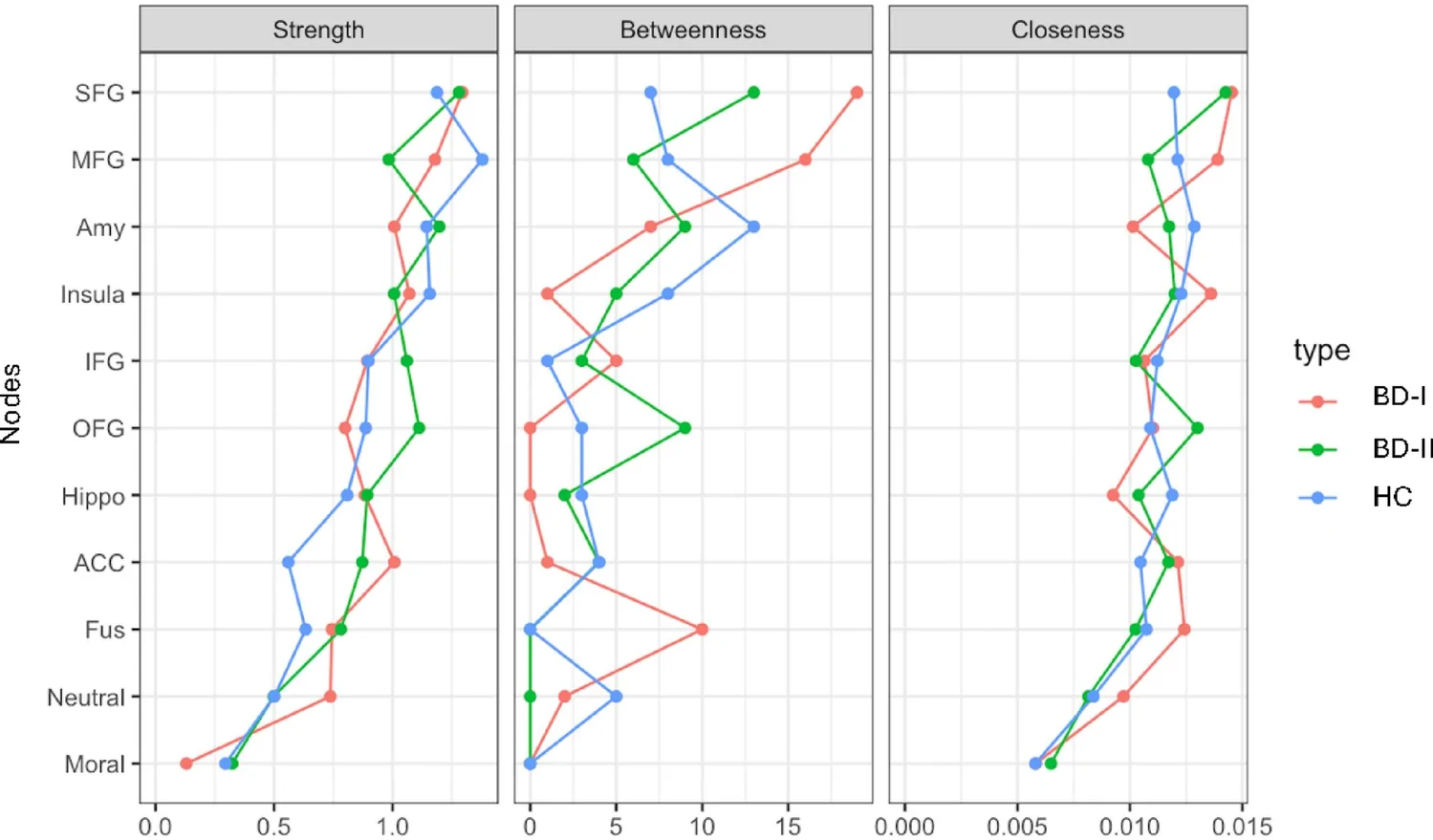
Centrality measures of the study variables for BD-I (in red), BD-II (in green), and HC (in blue). On the x-axis: the value of the centrality measures (Strength, Betweenness, Closeness). On the y-axis: FER-moral scores, FER-neutral score, Brain morphometry of the regions implicated in facial emotion recognition (nodes)

Centrality analyses revealed that in patients with BD-I, SFG showed the highest betweenness (0.422) and also ranked first for strength (1.295) and closeness (0.145), followed by MFG (betweenness 0.356; strength 1.179; closeness 0.139) and Fusiform (betweenness 0.222), with Insula also highly central by strength (1.071) and closeness (0.136). In BD‑II, SFG remained prominent (betweenness 0.289; strength 1.281; closeness 0.143), and both Amygdala and OFG showed elevated centrality (Amygdala: strength 1.198, betweenness 0.200, closeness 0.117; OFG: strength 1.112, betweenness 0.200, closeness 0.130). In HC, limbic nodes dominated non‑degree metrics, Amygdala (betweenness 0.289, closeness 0.129) and Insula (betweenness 0.178, closeness 0.123), while frontal hubs retained the highest strength (MFG 1.379, SFG 1.187; closeness 0.121 and 0.120, respectively) (Fig. 3).

Centrality-stability coefficients were strength: HC 0.40, BD-II 0.14, BD-I 0; closeness ≤ 0.20; betweenness 0; Supplementary Table S1). Bootstrapped 95% edge-weight confidence intervals identified stable edges (CI excluding zero) in HC and BD-II but none in BD-I (Supplementary Figure S3, Table S3).

## Discussion

This study investigated moral facial emotion recognition and its structural brain correlates in euthymic BD. Three main findings emerged. First, behaviorally, BD-I showed lower FER-moral performance than both BD-II and HC, whereas BD-II and HC did not differ; by contrast FER-neutral performance was comparable across groups. Second, while the overall strength of the FER-GMV networks did not differ across diagnostic groups, the relationship between nodes showed a subtype-specific pattern: BD-II differed from HC at the whole-network level, whereas BD-I showed, in exploratory analyses, a more frontally weighted centrality profile and edge-level differences in fronto–limbic structural covariance, particularly involving the amygdala, insula, ACC, and superior/middle frontal gyri.

The behavioral findings of this study indicate that impaired decoding of morally salient facial cues may be more characteristic of BD-I than BD-II. Although moral expressions were identified less efficiently than neutral expressions in the whole sample, this reduction was more evident in BD-I, consistent with evidence that FER deficits for negative emotions may persist during euthymia and may represent a trait-related feature of BD [2, 9]. The lack of between-group differences for neutral expressions further supports that the deficit is not a generalized slowing or nonspecific task failure, but rather reflects impaired processing of socially salient negative cues. Given that facial expressions of anger and disgust signal norm violations and social disapproval, reduced recognition of these expressions may contribute to the interpersonal difficulties often observed in BD-I. Consistent with this interpretation, Carpentier et al. reported impaired FER in euthymic BD, particularly for anger and contempt [56]. More broadly, social dysfunction in BD-I has been described as reduced conversational/social confidence and lower openness to new social situations in euthymic adults [57], while pediatric BD-I samples show greater peer difficulties associated with poorer Theory of Mind performance [58].

At the network level, global strength was similar across groups, whereas network structure differed only between BD-II and HC. By contrast, the significant network-structure difference indicates a different configuration of edges, consistent with a selective reconfiguration of brain-behavior and inter-regional couplings rather than a generalized increase or decrease in overall connectivity. Importantly, given the lack of whole-network differences in the BD-I comparisons, our data cannot support a generalized network-level alteration in BD-I. Instead, BD-I appears to be characterized by a more circumscribed shift in influential nodes and specific fronto-limbic couplings, as reflected in the centrality and edge-level findings. This interpretation is broadly consistent with prior works showing altered network organization and brain–behavior coupling in euthymic BD, including reduced frontal-insular-occipital covariance and abnormal amygdala-FER coupling [20, 23, 59].

The edge-specific results further support the presence of subtype-specific brain-behavior coupling. Most notably, the association between FER-neutral and amygdala GMV showed different directions across groups, being near-zero or weakly positive in BD-I and HC, and negative in BD-II, respectively. This pattern suggests that differences in amygdala-related structural covariance across BD subtypes may influence the processing of even low-salience social cues. This pattern is broadly consistent with prior functional evidence of subtype differences in fronto–amygdala regulation, with stronger inverse DLPFC–amygdala coupling in BD-II than BD-I [60].

Additional differences involving the amygdala, insula, ACC, and prefrontal regions indicate that the function of salience and executive network regions varies across bipolar phenotypes [27].

The centrality analyses refine this interpretation. Although BD-I did not differ from HC on the global network structure test, its internal hierarchy appeared, in exploratory analyses, more frontally weighted, with the SFG among the more central nodes, whereas in HC non-degree centrality was more concentrated in the amygdala and insula. One possible interpretation is that illness-related factors may be associated with a more frontally weighted pattern of fronto–limbic structural covariance in BD-I. Since structural covariance reflects coordinated inter-individual variation in gray-matter volume rather than online neural activity, this pattern cannot be interpreted as compensation, regulation, or altered “recruitment” but only as correlational; it is descriptively consistent with the altered fronto–limbic organisation reported in functional studies of euthymic BD-I [61–64]. At the same time, since structural covariance does not index online neural regulation and global invariance tests may be less sensitive to localized reorganization in relatively small samples, the absence of a significant whole-network effect in BD-I should not be taken as evidence of preserved network normality, especially in light of prior reports of altered connectome organization in BD-I [65]. However, in the absence of direct BD-I versus BD-II network-structure differences, distinctions between subtypes should be interpreted with caution; at most, the present findings are consistent with the broader evidence that BD-I and BD-II should not be treated as entirely homogeneous conditions [5, 6].

BD-II showed a different emotional processing pattern from BD-I. Behavioral performance was preserved, yet the network structure differed significantly from HC and included a distinct FER-neutral-amygdala coupling pattern together with altered fronto-limbic edges. BD-II may show an alternative configuration of structural covariance that co-occurs with preserved task performance without reproducing the normative topology observed in controls. Our data are in line with prior work suggesting that intact behavioral performance in BD can coexist with altered recruitment or organization of socio-emotional circuitry [66, 67]. However, the present design does not allow us to establish whether this pattern reflects compensatory, adaptive, or phenotypically distinct mechanisms.

Regarding the clinical and the conceptual implications, two practical points follow. First, moral emotion FER appears sensitive to subtype differences, with a measurable deficit in BD-I and preserved performance in BD-II. Second, network findings pinpoint candidate structural substrates (SFG, MFG, insula, amygdala, ACC) whose GMV covariance patterns may index social cognitive vulnerability [3, 9]. Given the relevance of FER to real-world functioning, targeting moral‑emotion decoding (alongside Theory‑of‑Mind work) may yield meaningful interpersonal benefits [3, 68].

However, several limitations must be acknowledged. First, the cross-sectional design does not allow causal inferences about the direction of brain–behavior relationships; the present study was intended to characterize subtype-specific association patterns, whereas longitudinal work is needed to test temporal dynamics across mood states. Second, medication may confound the subtype comparison: antipsychotic exposure was higher in BD-I and correlated with poorer FER-moral, and the subtype difference was attenuated after adjusting for medication. Given the incomplete dosage data and the strong collinearity between subtype and antipsychotic use, medication and subtype cannot be disentangled, and antipsychotic exposure remains an important potential confound. Sensitivity analysis adjusting for medication were limited by missing data and should be interpreted with caution. Replication in larger, medication-balanced samples is warranted. Participants were nonetheless euthymic and on stable regimens which increases ecological validity. Third, sample size limits generalizability and may reduce sensitivity to subtle effects; therefore, replication in larger independent cohorts is warranted, ideally integrating more ecologically valid socio-emotional paradigms. Fourth, Network analyses in the diagnostic subgroups, particularly in BD-I, should be regarded as exploratory and hypothesis-generating. Since the centrality-stability coefficients did not reach the recommended thresholds for stable Gaussian graphical model estimation, centrality indexes are interpreted as hypothesis-generating findings, and the more frontally weighted BD-I profile requires replication in larger samples; likewise, edge-level differences other than the one surviving correction should be treated as preliminary. To strengthen and validate these observations, future research should include longitudinal designs to track moral FER performance across mood states, integrate functional and neurochemical imaging data to dissect underlying mechanisms, and adopt more ecologically valid paradigms (e.g., dynamic or contextualized social cues) to better capture moral emotion processing in real-life interactions. Of note, these findings are based on structural covariance rather than functional activation, thus they cannot be interpreted as direct evidence of impaired top-down regulation.

In conclusion, moral emotion recognition and its structural correlates showed subtype-specific patterns in BD. BD-I was characterized by impaired decoding of morally salient facial expressions alongside exploratory, more frontally weighted differences in fronto–limbic structural covariance, rather than a global network disruption. In contrast, BD-II exhibited preserved behavioral performance despite altered network topology, consistent with a distinct pattern of brain–behavior organization.

The present findings support emerging evidence that BD-I and BD-II show partially distinct neurocognitive and neurobiological profiles, particularly in affective and social-cognitive domains. Importantly, moral emotion recognition may represent a sensitive and clinically relevant marker, while network-level metrics provide complementary insights into underlying neural organization.

From a translational perspective, these results highlight fronto–limbic circuits as potential targets for interventions aimed at improving social functioning and suggest that subtype-specific approaches may be required to optimize treatment strategies in individuals living with BD.

## Supplementary Information

Below is the link to the electronic supplementary material.

Supplementary Material 1

## Data Availability

The data that support the findings of this study are available upon reasonable request from the corresponding author.

## References

[1] Zhang B, Chen X, Qiu N (2025) Social cognition in bipolar I and II disorders: an updated systematic review and meta-analysis. BMC Psychiatry 25:39. 10.1186/S12888-024-06462-Z 39810149 PMC11734565

[2] Reddy PV, Anandan S, Rakesh G et al (2022) Emotion Processing Deficit in Euthymic Bipolar Disorder: A Potential Endophenotype. Indian J Psychol Med 44:145–151. 10.1177/02537176211026795 35655991 PMC9120978

[3] Vlad M, Raucher-Chéné D, Henry A, Kaladjian A (2018) Functional outcome and social cognition in bipolar disorder: Is there a connection? Eur Psychiatry 52:116–125. 10.1016/J.EURPSY.2018.05.002 29787961

[4] Miskowiak KW, Burdick KE, Martinez-Aran A et al (2018) Assessing and addressing cognitive impairment in bipolar disorder: the International Society for Bipolar Disorders Targeting Cognition Task Force recommendations for clinicians. Bipolar Disord 20:184–194. 10.1111/BDI.12595 29345040

[5] Simonsen C, Sundet K, Vaskinn A et al (2008) Neurocognitive profiles in bipolar I and bipolar II disorder: differences in pattern and magnitude of dysfunction. Bipolar Disord 10:245–255. 10.1111/J.1399-5618.2007.00492.X 18271903

[6] Tondo L, Miola A, Pinna M et al (2022) Differences between bipolar disorder types 1 and 2 support the DSM two-syndrome concept. Int J Bipolar Disord 10:1–11. 10.1186/S40345-022-00268-2 35918560 PMC9346033

[7] Bora E (2018) Neurocognitive features in clinical subgroups of bipolar disorder: A meta-analysis. J Affect Disord 229:125–134. 10.1016/j.jad.2017.12.057 29306692

[8] De Prisco M, Oliva V, Possidente C et al (2026) Facial emotion recognition deficits in bipolar disorder: a systematic review and meta-analysis. Eur Psychiatry 69:e16. 10.1192/J.EURPSY.2025.10147 41521787 PMC12925675

[9] Rocca CCDA, Van Den Heuvel E, Caetano SC, Lafer B (2009) Facial emotion recognition in bipolar disorder: a critical review. Revista brasileira de psiquiatria (Sao Paulo, Brazil : 1999) 31:171–180. 10.1590/S1516-44462009000200015 19578691

[10] Furlong LS, Rossell SL, Karantonis JA et al (2022) Characterization of facial emotion recognition in bipolar disorder: Focus on emotion mislabelling and neutral expressions. J Neuropsychol 16:353–372. 10.1111/JNP.12267 34762769

[11] van der Eijk F, Columbus S (2023) Expressions of moral disgust reflect both disgust and anger. Cogn Emot 37:499–514. 10.1080/02699931.2023.2183179 36864728

[12] Rozin P, Lowery L, Imada S, Haidt J (1999) The CAD triad hypothesis: A mapping between three moral emotions (contempt, anger, disgust) and three moral codes (community, autonomy, divinity). J Pers Soc Psychol 76:574–586. 10.1037/0022-3514.76.4.574 10234846

[13] Haidt J (2003) The Moral Emotions. In: Davidson RJ, Scherer KR, Goldsmith HH (eds) Handbook of Affective Sciences. Oxford University Press, Oxford, pp 852–870

[14] Bzdok D, Schilbach L, Vogeley K et al (2012) Parsing the neural correlates of moral cognition: ALE meta-analysis on morality, theory of mind, and empathy. Brain Struct Funct 217:783–796. 10.1007/S00429-012-0380-Y 22270812 PMC3445793

[15] Moll J, De Oliveira-Souza R, Eslinger PJ et al (2002) The neural correlates of moral sensitivity: a functional magnetic resonance imaging investigation of basic and moral emotions. J Neurosci 22:2730. 10.1523/JNEUROSCI.22-07-02730.2002 11923438 PMC6758288

[16] Moll J, De Oliveira-Souza R, Moll FT et al (2005) The moral affiliations of disgust: a functional MRI study. Cogn Behav Neurol 18:68–78. 10.1097/01.WNN.0000152236.46475.A7 15761278

[17] Fede SJ, Kiehl KA (2020) Meta-analysis of the moral brain: patterns of neural engagement assessed using multilevel kernel density analysis. Brain Imaging Behav 14:534–547. 10.1007/S11682-019-00035-5 30706370

[18] Neves M, Albuquerque MR, Malloy-Diniz L et al (2015) A voxel-based morphometry study of gray matter correlates of facial emotion recognition in bipolar disorder. Psychiatry Res 233:158–164. 10.1016/J.PSCYCHRESNS.2015.05.009 26123449

[19] Hibar DP, Westlye LT, Van Erp TGM et al (2016) Subcortical volumetric abnormalities in bipolar disorder. Mol Psychiatry 21:1710–1716. 10.1038/MP.2015.227 26857596 PMC5116479

[20] Miola A, Trevisan N, Merola A et al (2022) Gray matter volume covariance networks are associated with altered emotional processing in bipolar disorder: a source-based morphometry study. Brain Imaging Behav 16:738–747. 10.1007/S11682-021-00541-5 34546520 PMC9010334

[21] Bora E, Fornito A, Yücel M, Pantelis C (2010) Voxelwise Meta-Analysis of Gray Matter Abnormalities in Bipolar Disorder. Biol Psychiatry 67:1097–1105. 10.1016/J.BIOPSYCH.2010.01.020 20303066

[22] Ellison-Wright I, Bullmore E (2010) Anatomy of bipolar disorder and schizophrenia: A meta-analysis. Schizophr Res 117:1–12. 10.1016/J.SCHRES.2009.12.022 20071149

[23] Miola A, Trevisan N, Salvucci M et al (2024) Network dysfunction of sadness facial expression processing and morphometry in euthymic bipolar disorder. Eur Arch Psychiatry Clin Neurosci 274:525–536. 10.1007/S00406-023-01649-Z 37498325 PMC10995000

[24] Bringmann LF, Elmer T, Epskamp S et al (2019) What do centrality measures measure in psychological networks? J Abnorm Psychol 128:892–903. 10.1037/ABN0000446 31318245

[25] Epskamp S, Borsboom D, Fried EI (2018) Estimating psychological networks and their accuracy: a tutorial paper. Behav Res Methods 50:195–212. 10.3758/S13428-017-0862-1 28342071 PMC5809547

[26] Cusi AM, Nazarov A, Holshausen K et al (2012) Systematic review of the neural basis of social cognition in patients with mood disorders. J Psychiatry Neurosci 37:154. 10.1503/JPN.100179 22297065 PMC3341408

[27] Miola A, Salvucci M, Meda N et al (2026) Neural signatures of bipolar disorder subtypes: a comprehensive systematic review of neuroimaging studies. J Affect Disord 394:120605 10.1016/J.JAD.2025.120605 41192728

[28] Lahera G, Benito A, Montes JM et al (2013) Social cognition and interaction training (SCIT) for outpatients with bipolar disorder. J Affect Disord 146:132–136. 10.1016/J.JAD.2012.06.032 22840617

[29] World Medical Association (2025) World Medical Association Declaration of Helsinki: Ethical Principles for Medical Research Involving Human Participants. JAMA 333:71–74. 10.1001/JAMA.2024.21972 39425955

[30] Montgomery SA, Asberg M (1979) A new depression scale designed to be sensitive to change. Br J Psychiatry 134:382–389. 10.1192/BJP.134.4.382 444788

[31] Hamilton M (1960) A rating scale for depression. J Neurol Neurosurg Psychiatry 23:56–62. 10.1136/JNNP.23.1.56 14399272 PMC495331

[32] Hamilton M (1959) The assessment of anxiety states by rating. Br J Med Psychol 32:50–55. 10.1111/J.2044-8341.1959.TB00467.X 13638508

[33] Young RC, Biggs JT, Ziegler VE, Meyer DA (1978) A rating scale for mania: reliability, validity and sensitivity. Br J Psychiatry 133:429–435. 10.1192/BJP.133.5.429 728692

[34] Kay SR, Fiszbein A, Opler LA (1987) The positive and negative syndrome scale (PANSS) for schizophrenia. Schizophr Bull 13:261–276. 10.1093/SCHBUL/13.2.261 3616518

[35] Aas IHM (2011) Guidelines for rating Global Assessment of Functioning (GAF). Ann Gen Psychiatry 10:1–11. 10.1186/1744-859X-10-2 21251305 PMC3036670

[36] Nosè M, Barbui C (2008) A simple approach to manage dosages in drug-epidemiology research. Epidemiol Psichiatr Soc 17:186–187. 10.1017/S1121189X00001263 18924556

[37] De Panfilis C, Antonucci C, Meehan KB et al (2019) Facial emotion recognition and social-cognitive correlates of narcissistic features. J Pers Disord 33:433–449. 10.1521/PEDI_2018_32_350 29847219

[38] Meehan KB, De Panfilis C, Cain NM et al (2017) Facial emotion recognition and borderline personality pathology. Psychiatry Res 255:347–354. 10.1016/j.psychres.2017.05.042 28605709

[39] Vandierendonck A (2017) A comparison of methods to combine speed and accuracy measures of performance: A rejoinder on the binning procedure. Behav Res Methods 49:653–673. 10.3758/S13428-016-0721-5 26944576

[40] Leathem LD, Currin DL, Montoya AK, Karlsgodt KH (2021) Socioemotional mechanisms of loneliness in subclinical psychosis. Schizophr Res 238:145–151. 10.1016/j.schres.2021.10.002 34688116 PMC8896506

[41] Gan X, Zhang R, Zheng Z et al (2025) Does unfairness evoke anger or disgust? A quantitative neurofunctional dissection based on 25 years of neuroimaging. Neurosci Biobehav Rev 178:106356. 10.1016/J.NEUBIOREV.2025.106356 40914417

[42] Gur RC, Schroeder L, Turner T et al (2002) Brain Activation during Facial Emotion Processing. NeuroImage 16:651–662. 10.1006/NIMG.2002.1097 12169250

[43] Haxby JV, Hoffman EA, Gobbini MI (2002) Human neural systems for face recognition and social communication. Biol Psychiatry 51:59–67. 10.1016/S0006-3223(01)01330-0 11801231

[44] Purves D, Augustine GJ, Fitzpatrick D (2001) Neuroscience. Sinauer Associates, MA, USA

[45] Femenía T, Gómez-Galán M, Lindskog M, Magara S (2012) Dysfunctional hippocampal activity affects emotion and cognition in mood disorders. Brain Res 1476:58–70. 10.1016/J.BRAINRES.2012.03.053 22541166

[46] Craig AD (2009) How do you feel—now? The anterior insula and human awareness. Nat Rev Neurosci. 10.1038/nrn2555 19096369

[47] Rolls ET (2019) The cingulate cortex and limbic systems for emotion, action, and memory. Brain Struct Funct 224:3001–3018. 10.1007/S00429-019-01945-2 31451898 PMC6875144

[48] Kesler-West ML, Andersen AH, Smith CD et al (2001) Neural substrates of facial emotion processing using fMRI. Cogn Brain Res 11:213–226. 10.1016/S0926-6410(00)00073-2 11275483

[49] Beer JS, Knight RT, D’Esposito M (2006) Controlling the Integration of Emotion and Cognition. Psychol Sci 17:448–453. 10.1111/J.1467-9280.2006.01726.X 16683934

[50] R Core Team, R Core Team (2022) (2022). R: A language and environment for statistical computing. R Foundation for Statistical Computing, Vienna, Austria. URL https://www.R-project.org/

[51] Epskamp S, Cramer AOJ, Waldorp LJ et al (2012) qgraph: Network Visualizations of Relationships in Psychometric Data. J Stat Softw 48:1–18. 10.18637/jss.v048.i04

[52] Epskamp S, Fried EI (2018) A Tutorial on Regularized Partial Correlation Networks. Psychol Methods 23:617–634. 10.1037/met0000167 29595293

[53] Chen J, Chen Z (2008) Extended Bayesian information criteria for model selection with large model spaces. Biometrika 95:759–771. 10.1093/BIOMET/ASN034

[54] Tibshirani R (1996) Regression Shrinkage and Selection via the Lasso. J Royal Stat Soc Ser B (Methodological) 58:267–288

[55] Smith KE, Mason TB, Crosby RD et al (2019) A comparative network analysis of eating disorder psychopathology and co-occurring depression and anxiety symptoms before and after treatment. Psychol Med 49:314–324. 10.1017/S0033291718000867 29655386 PMC6310232

[56] Carpentier A, Angerville B, Delille S et al (2025) Impairments in facial expression recognition in patients with euthymic bipolar disorders using the facial emotions recognition test. J Affect Disord 370:241–248. 10.1016/J.JAD.2024.11.016 39515481

[57] Castanho de Almeida Rocca C, Britto de Macedo-Soares M, Gorenstein C et al (2008) Social dysfunction in bipolar disorder: pilot study. Aust N Z J Psychiatry 42:686–692. 10.1080/00048670802203426 18622776

[58] Schenkel LS, Chamberlain TF, Towne TL (2014) Impaired Theory of Mind and psychosocial functioning among pediatric patients with Type I versus Type II bipolar disorder. Psychiatry Res 215:740–746. 10.1016/J.PSYCHRES.2013.10.025 24461271

[59] Dvorak J, Hilke M, Trettin M et al (2019) Aberrant brain network topology in fronto-limbic circuitry differentiates euthymic bipolar disorder from recurrent major depressive disorder. Brain Behav. 10.1002/BRB3.1257 31066228 PMC6576154

[60] Caseras X, Murphy K, Lawrence NS et al (2015) Emotion regulation deficits in euthymic bipolar I versus bipolar II disorder: a functional and diffusion-tensor imaging study. Bipolar Disord 17:461–470. 10.1111/BDI.12292 25771686 PMC4672703

[61] Corbalán F, Beaulieu S, Armony JL (2015) Emotion regulation in bipolar disorder type I: an fMRI study. Psychol Med 45:2521–2531. 10.1017/S0033291715000434 25997918

[62] Mesbah R, Koenders MA, Van Der Wee NJA et al (2023) Association between the fronto-limbic network and cognitive and emotional functioning in individuals with bipolar disorder: a systematic review and meta-analysis. JAMA Psychiatry 80:432. 10.1001/JAMAPSYCHIATRY.2023.0131 36988918 PMC10061312

[63] Radaelli D, Sferrazza Papa G, Vai B et al (2015) Fronto-limbic disconnection in bipolar disorder. Eur Psychiatry 30:82–88. 10.1016/J.EURPSY.2014.04.001 24853295

[64] Townsend JD, Torrisi SJ, Lieberman MD et al (2012) Frontal-amygdala connectivity alterations during emotion down-regulation in bipolar I disorder. Biol Psychiatry 73:127–135. 10.1016/J.BIOPSYCH.2012.06.030 22858151 PMC3525751

[65] Collin G, van den Heuvel MP, Abramovic L et al (2016) Brain network analysis reveals affected connectome structure in bipolar I disorder. Hum Brain Mapp 37:122–134. 10.1002/HBM.23017 26454006 PMC5597048

[66] Bertocci MA, Bergman J, Santos JPL et al (2020) Emotional regulation neural circuitry abnormalities in adult bipolar disorder: dissociating effects of long-term depression history from relationships with present symptoms. Transl Psychiatry 10:374. 10.1038/S41398-020-01048-1 33139703 PMC7608654

[67] Hwang HC, Kim SM, Han DH (2021) Different facial recognition patterns in schizophrenia and bipolar disorder assessed using a computerized emotional perception test and fMRI. J Affect Disord 279:83–88. 10.1016/j.jad.2020.09.125 33039778

[68] Mitchell RLC, Young AH (2016) Theory of Mind in Bipolar Disorder, with Comparison to the Impairments Observed in Schizophrenia. Front Psychiatry 6:188. 10.3389/FPSYT.2015.00188 26834648 PMC4716141

