# Supplementary material for: Moral emotion processing in euthymic bipolar disorder: insights from network analysis
Source: Eur Arch Psychiatry Clin Neurosci. 2026 Aug 8;276(7):3803–14. doi: 10.1007/s00406-026-02344-5 (PMC13562291; doi:10.1007/s00406-026-02344-5)
Supplement: Supplementary file 1 [file 406_2026_2344_MOESM1_ESM.docx]

**Supplementary Material to:**

**Moral Emotion Processing in Euthymic Bipolar Disorder: Insights from Network Analysis**

Marialaura Lussignoli ^1,2^, Giulia Cattarinussi ^1,3^, Alessandro Miola ^1,4^, Nicolò Trevisan ^1,2^, Silvia Valeggia ^1,5^, Renzo Manara ^1,5^, Fabio Sambataro ^1,2**^

^1^ Department of Neuroscience (DNS), University of Padova, Padua, Italy.

^2^ Padova Neuroscience Center, University of Padova, Padua, Italy.

^3^ Department of Psychological Medicine, Institute of Psychiatry, Psychology and Neuroscience, King's College London, London, UK.

^4^ Department of Psychiatry & Psychology, Mayo Clinic, Rochester, Minnesota, USA.

^5^ Neuroradiology Unit, Department of Neurosciences, University of Padova, Padova, Italy.

**Corresponding author:

Prof. Fabio Sambataro, M.D., Ph.D.

Department of Neuroscience, Via Giustiniani, 2, 35128 Padova, Italy;

Tel. +39 0498211980; Email: Fabio.sambataro@unipd.it

## Table S1. Centrality-stability (CS) coefficients

*Maximum proportion of cases that can be dropped while retaining a correlation ≥ 0.70 with the original centrality in ≥95% of subsamples (case-dropping bootstrap, bootnet). Values ≥ 0.50 are considered interpretable.*

| **Group** | **Strength** | **Closeness** | **Betweenness** |
| --- | --- | --- | --- |
| HC (n=40) | 0.40 | 0.20 | 0 |
| BD-II (n=28) | 0.14 | 0 | 0 |
| BD-I (n=19) | 0 | 0 | 0 |

All coefficients are below 0.50; betweenness is unstable in every group and BD-I is unstable across all indices. Centrality is therefore interpreted descriptively only.

## Table S2. Edge-level comparisons: uncorrected and FDR-corrected p-values

*Network Comparison Test (independent groups, 5,000 permutations), all 55 edges tested per comparison. Only edges with uncorrected p < 0.05 are listed; Benjamini–Hochberg correction was applied across all 55 edges of each comparison.*

### BD-I vs BD-II

| **Edge** | **p (uncorrected)** | **p (FDR)** | **Survives FDR** |
| --- | --- | --- | --- |
| ACC – MFG | 0.0110 | 0.506 | no |
| ACC – SFG | 0.0210 | 0.506 | no |
| Amy – Insula | 0.0338 | 0.506 | no |
| Insula – SFG | 0.0396 | 0.506 | no |
| Neutral – Amy | 0.0460 | 0.506 | no |

### BD-I vs HC

| **Edge** | **p (uncorrected)** | **p (FDR)** | **Survives FDR** |
| --- | --- | --- | --- |
| Insula – ACC | 0.0430 | 0.841 | no |
| Insula – SFG | 0.0462 | 0.841 | no |

### BD-II vs HC

| **Edge** | **p (uncorrected)** | **p (FDR)** | **Survives FDR** |
| --- | --- | --- | --- |
| Insula – IFG | 0.0006 | 0.033 | **yes** |
| MFG – IFG | 0.0028 | 0.077 | no |
| ACC – MFG | 0.0138 | 0.228 | no |
| Neutral – Amy | 0.0166 | 0.228 | no |
| ACC – SFG | 0.0226 | 0.249 | no |

*Neutral, FER-neutral; Amy, amygdala; Insula; ACC, anterior cingulate cortex; MFG/IFG/SFG, middle/ inferior/superior frontal gyrus*

**Table S3. Partial-correlation edge weights with bootstrapped 95% confidence intervals, by group**

*Edges with a non-zero weight in the regularized partial-correlation (EBICglasso) network are listed for each group, ordered by absolute weight. Partial r is the edge weight (partial correlation); 95% CI is the non-parametric bootstrap confidence interval. * indicates edges whose 95% CI excludes zero. Node abbreviations: Moral/Neutral, FER-moral/FER-neutral; Hippo, hippocampus; Amy, amygdala; Fus, fusiform gyrus; Insula; ACC, anterior cingulate cortex; MFG/OFG/IFG/SFG, middle/orbital/inferior/superior frontal gyrus.*

**BD-I (n=19)**

| **Edge** | **Partial r** | **95% CI** |  |
| --- | --- | --- | --- |
| Hippo – Amy | +0.638 | [+0.000, +0.801] |  |
| ACC – MFG | +0.409 | [+0.000, +0.619] |  |
| Insula – SFG | +0.352 | [+0.000, +0.547] |  |
| Neutral – IFG | +0.333 | [+0.000, +0.633] |  |
| MFG – SFG | +0.323 | [+0.000, +0.456] |  |
| Insula – ACC | +0.282 | [+0.000, +0.500] |  |
| Fus – SFG | +0.279 | [+0.000, +0.528] |  |
| Amy – Fus | +0.248 | [+0.000, +0.383] |  |
| MFG – IFG | +0.236 | [+0.000, +0.606] |  |
| MFG – OFG | +0.210 | [+0.000, +0.388] |  |
| Neutral – ACC | -0.205 | [-0.504, +0.003] |  |
| Fus – OFG | +0.195 | [+0.000, +0.447] |  |
| Insula – OFG | +0.180 | [+0.000, +0.435] |  |
| Insula – IFG | +0.150 | [+0.000, +0.311] |  |
| OFG – SFG | +0.116 | [-0.009, +0.315] |  |
| Hippo – Insula | +0.107 | [+0.000, +0.348] |  |
| Neutral – Amy | +0.101 | [-0.075, +0.297] |  |
| Moral – SFG | +0.087 | [+0.000, +0.366] |  |
| ACC – OFG | +0.076 | [+0.000, +0.347] |  |
| Neutral – Hippo | +0.064 | [-0.102, +0.360] |  |
| IFG – SFG | +0.052 | [+0.000, +0.405] |  |
| Hippo – SFG | +0.051 | [+0.000, +0.176] |  |
| Moral – IFG | +0.043 | [-0.130, +0.541] |  |
| ACC – IFG | +0.036 | [-0.035, +0.324] |  |
| Neutral – SFG | +0.035 | [+0.000, +0.364] |  |
| OFG – IFG | +0.024 | [+0.000, +0.459] |  |
| Hippo – Fus | +0.022 | [-0.082, +0.431] |  |
| Amy – IFG | -0.021 | [-0.278, +0.077] |  |

**BD-II (n=28)**

| **Edge** | **Partial r** | **95% CI** |  |
| --- | --- | --- | --- |
| MFG – IFG | +0.617 | [+0.269, +0.720] | * |
| Hippo – Amy | +0.565 | [+0.314, +0.724] | * |
| ACC – SFG | +0.402 | [+0.119, +0.566] | * |
| Insula – OFG | +0.307 | [+0.061, +0.475] | * |
| MFG – SFG | +0.243 | [+0.029, +0.406] | * |
| Fus – Insula | +0.209 | [+0.000, +0.452] |  |
| Amy – Insula | +0.204 | [+0.000, +0.362] |  |
| OFG – SFG | +0.200 | [+0.010, +0.322] | * |
| OFG – IFG | +0.187 | [+0.019, +0.385] | * |
| Fus – SFG | +0.173 | [+0.000, +0.316] |  |
| Amy – SFG | +0.154 | [+0.000, +0.332] |  |
| Neutral – OFG | -0.151 | [-0.396, +0.000] |  |
| ACC – OFG | +0.146 | [+0.000, +0.339] |  |
| Neutral – Hippo | +0.119 | [+0.000, +0.409] |  |
| ACC – IFG | +0.119 | [+0.000, +0.399] |  |
| Insula – ACC | +0.102 | [+0.000, +0.321] |  |
| Moral – ACC | -0.101 | [-0.377, +0.000] |  |
| Hippo – Fus | +0.098 | [-0.100, +0.395] |  |
| Amy – Fus | +0.097 | [+0.000, +0.375] |  |
| Neutral – Amy | -0.096 | [-0.332, +0.000] |  |
| Moral – Amy | +0.082 | [-0.041, +0.325] |  |
| Neutral – Insula | +0.082 | [-0.081, +0.434] |  |
| IFG – SFG | +0.077 | [+0.000, +0.404] |  |
| Hippo – MFG | +0.069 | [+0.000, +0.205] |  |
| Moral – Fus | -0.068 | [-0.367, +0.150] |  |
| Fus – IFG | +0.061 | [-0.017, +0.191] |  |
| MFG – OFG | +0.054 | [+0.000, +0.262] |  |
| Neutral – Fus | +0.050 | [-0.077, +0.320] |  |
| Moral – OFG | +0.043 | [-0.180, +0.371] |  |
| Hippo – Insula | +0.041 | [+0.000, +0.305] |  |
| Insula – SFG | +0.031 | [+0.000, +0.200] |  |
| Moral – Insula | +0.030 | [-0.116, +0.240] |  |
| Fus – OFG | +0.023 | [+0.000, +0.246] |  |
| Fus – ACC | +0.003 | [+0.000, +0.236] |  |

**HC (n=40)**

| **Edge** | **Partial r** | **95% CI** |  |
| --- | --- | --- | --- |
| MFG – SFG | +0.471 | [+0.300, +0.587] | * |
| Hippo – Amy | +0.470 | [+0.210, +0.647] | * |
| Insula – IFG | +0.424 | [+0.274, +0.536] | * |
| ACC – MFG | +0.336 | [+0.134, +0.484] | * |
| Insula – OFG | +0.301 | [+0.116, +0.488] | * |
| Amy – Fus | +0.275 | [+0.020, +0.414] | * |
| Amy – Insula | +0.241 | [+0.000, +0.389] |  |
| MFG – OFG | +0.229 | [+0.000, +0.385] |  |
| Hippo – SFG | +0.184 | [+0.005, +0.350] | * |
| Fus – SFG | +0.183 | [+0.004, +0.304] | * |
| OFG – SFG | +0.182 | [+0.006, +0.311] | * |
| ACC – IFG | +0.155 | [+0.000, +0.294] |  |
| OFG – IFG | +0.130 | [+0.000, +0.367] |  |
| Moral – Neutral | +0.130 | [-0.006, +0.386] |  |
| Neutral – Amy | +0.123 | [+0.000, +0.330] |  |
| Insula – MFG | +0.111 | [+0.000, +0.235] |  |
| Neutral – IFG | +0.095 | [-0.026, +0.287] |  |
| Fus – MFG | +0.091 | [+0.000, +0.264] |  |
| Neutral – SFG | -0.078 | [-0.269, +0.000] |  |
| Hippo – Fus | +0.074 | [+0.000, +0.308] |  |
| Neutral – MFG | -0.073 | [-0.225, +0.000] |  |
| Moral – ACC | +0.069 | [-0.030, +0.315] |  |
| MFG – IFG | +0.068 | [+0.000, +0.290] |  |
| Insula – SFG | +0.055 | [+0.000, +0.173] |  |
| Hippo – OFG | +0.045 | [+0.000, +0.179] |  |
| Amy – SFG | +0.035 | [+0.000, +0.270] |  |
| Moral – Hippo | -0.034 | [-0.182, +0.025] |  |
| Moral – IFG | +0.027 | [-0.153, +0.336] |  |
| Moral – Insula | +0.025 | [+0.000, +0.245] |  |
| Moral – Fus | -0.011 | [-0.221, +0.000] |  |
| Neutral – Hippo | +0.002 | [-0.045, +0.232] |  |

**Table S4. FER-moral group comparison with covariates**

*One-way model and covariate-adjusted models for FER-moral (efficiency). Group omnibus F/p and adjusted pairwise p-values. Education differed across groups (F=7.82, p=0.0008).*

| **Model** | **N** | **df** | **F (group)** | **BD-I vs BD-II** | **BD-I vs HC** | **BD-II vs HC** |
| --- | --- | --- | --- | --- | --- | --- |
| Unadjusted | 87 | 2, 84 | 5.93 (p=.004) | 0.020 | 0.004 | 0.887 |
| + sex + education | 87 | 2, 82 | 4.42 (p=.015) | 0.008 | 0.011 | 0.893 |
| + sex + education + age | 87 | 2, 81 | 3.43 (p=.037) | 0.044 | 0.013 | 0.639 |

The BD-I deficit in FER-moral was robust to adjustment for sex, education, and age.

## Table S5. Medication exposure and FER-moral (sensitivity analysis)

*Per-subject Defined Daily Dose (DDD) for antipsychotics (AP) and antidepressants (AD) and lithium units, in the BD subsample with available data (n=28–30).*

### A. DDD by subtype (mean ± SD)

| **Drug class** | **BD-I** | **BD-II** | **p** |
| --- | --- | --- | --- |
| Antipsychotics (DDD) | 0.95 ± 0.94 (n=10) | 0.17 ± 0.37 (n=18) | 0.030 |
| Antidepressants (DDD) | 0.35 ± 0.71 (n=12) | 0.82 ± 0.79 (n=18) | 0.107 |
| Lithium (units) | 1.62 ± 0.83 (n=11) | 1.78 ± 0.31 (n=18) | 0.542 |

### B. Correlation of DDD with FER-moral (within BD, Spearman)

| **Variable** | **ρ** | **p** |
| --- | --- | --- |
| Antipsychotics (DDD) | −0.42 | 0.028 |
| Antidepressants (DDD) | +0.38 | 0.038 |
| Lithium (units) | +0.44 | 0.018 |

### C. BD-I vs BD-II FER-moral, adjusting for medication

| **Model** | **N** | **p (group)** |
| --- | --- | --- |
| Unadjusted | 47 | 0.003 |
| + antipsychotic + antidepressant DDD | 28 | 0.82 |
| + AP + AD + lithium | 28 | 0.72 |

Antipsychotic exposure was higher in BD-I and correlated with poorer FER-moral; the subtype difference was attenuated after adjustment.

**Table S6. FER accuracy and reaction time by group**

*Response accuracy (percent correct) and mean reaction time (mean ± SD) for moral (anger + disgust) and neutral expressions, and overall (total), by diagnostic group. F and p: one-way ANOVA across the three groups (between-groups df = 2, residual df = 76). Trial-level data were available for 79 participants (BD-I 17, BD-II 26, HC 36).*

| **Measure** | **BD-I (n=17)** | **BD-II (n=26)** | **HC (n=36)** | ***F (2,76)*** | ***p*** |
| --- | --- | --- | --- | --- | --- |
| Accuracy – Moral (%) | 49.4 ± 9.2 | 54.7 ± 12.7 | 57.2 ± 11.9 | 2.56 | 0.084 |
| Accuracy – Neutral (%) | 88.5^a^ ± 12.3 | 90.4^ab^ ± 12.8 | 96.1^b^ ± 5.6 | 4.24 | 0.018 |
| Accuracy – Total (%) | 52.7^a^ ± 8.8 | 60.6^b^ ± 11.0 | 61.1^b^ ± 9.8 | 4.43 | 0.015 |
| Reaction time – Moral (ms) | 3937^a^ ± 1667 | 2833^b^ ± 594 | 3073^b^ ± 894 | 6.21 | 0.003 |
| Reaction time – Neutral (ms) | 2634^ab^ ± 937 | 2369^a^ ± 985 | 3033^b^ ± 1120 | 3.16 | 0.048 |

*Within each row, groups not sharing a superscript letter differ at p < 0.05 (Tukey HSD post-hoc).*

Overall accuracy was lower in BD-I than in BD-II and HC; for moral expressions, accuracy was lower in BD-I at a trend level and reaction times were significantly longer, indicating that the BD-I efficiency deficit reflects both reduced accuracy and slower responding.

# Supplementary Figures

### Figure S1. Centrality (strength, betweenness, closeness)

*Standardized centrality per group (type 1 = BD-I, type 2 = BD-II, type 3 = HC). Interpret descriptively (see Table S1).*

### Figure S2. Centrality-stability (case-dropping bootstrap)

*BD-I*

*BD-II*

*HC*

### Figure S3. Bootstrapped 95% CIs of edge weights

*BD-I — note wide CIs spanning zero (low stability).*

*BD-II*

*HC*
